# Identifying lncRNA- and Transcription Factor-Associated Regulatory Networks in the Cortex of Rats With Deep Hypothermic Circulatory Arrest

**DOI:** 10.3389/fgene.2021.746757

**Published:** 2021-12-17

**Authors:** Mengya Liang, Yi Zhang, Shuangjiao Gan, Yunqi Liu, Huayang Li, Quan Liu, Haoliang Liu, Zhuoming Zhou, Huawei Wu, Guangxian Chen, Zhongkai Wu

**Affiliations:** ^1^ Department of Cardiac Surgery, The First Affiliated Hospital, Sun Yat-Sen University, Guangzhou, China; ^2^ NHC Key Laboratory of Assisted Circulation, Sun Yat-Sen University, Guangzhou, China; ^3^ Department of Neurobiology, Physiology and Behavior, College of Biological Sciences, University of California, Davis, CA, United States

**Keywords:** deep hypothermic circulatory arrest (DHCA), high-throughput sequencing, long noncoding RNA (lncRNA), microRNA (miRNA), transcription factor (TF), transcription factor-miRNA coregulatory network (TF-miRNA coregulatory network)

## Abstract

Long noncoding RNAs (lncRNAs) and microRNAs (miRNAs) are involved in the mechanism underlying cerebral dysfunction after deep hypothermic circulatory arrest (DHCA), although the exact details have not been elucidated. To explore the expression profiles of lncRNAs and miRNAs in DHCA cerebral injury, we determined the lncRNA, miRNA and mRNA expression profiles in the cerebral cortex of DHCA and sham rats. First, a rat model of DHCA was established, and high-throughput sequencing was performed to analyze the differentially expressed RNAs (DERNAs). Then, the principal functions of the significantly deregulated genes were identified using Gene Ontology (GO) and Kyoto Encyclopedia of Genes and Genomes (KEGG) pathway enrichment analyses. Expression networks (lncRNAs-miRNAs-mRNAs and transcription factors (TFs)-miRNAs-mRNAs) were also established. Finally, the expression of DERNAs was confirmed by quantitative real-time PCR (RT-qPCR). We identified 89 lncRNAs, 45 miRNAs and 59 mRNAs between the DHCA and sham groups and constructed a comprehensive competitive endogenous RNAs (ceRNAs) network. A TF-miRNA-mRNA regulatory network was also established. Finally, we predicted that Lcorl-miR-200a-3p-Ttr, BRD4-Ccl2 and Ep300-miR-200b-3p-Tmem72 may participate in the pathogenesis of DHCA cerebral injury.

## 1 Introduction

Deep hypothermia circulatory arrest (DHCA) surgery has been more widely used in clinical practice in recent decades (Griepp et al., 1975). Compared with cardiopulmonary bypass (CPB), DHCA reduces the core temperature to 18°C, which can reduce the body metabolic rate and tissue oxygen consumption and improve organ tolerance to ischemia, thereby substantially reducing the risk of surgery. Hence, DHCA has become an important manipulation during complex cardiac surgery, such as aortic arch surgery and complex congenital heart disease correction. However, because the brain is extremely sensitive to hypoxia and ischemia/reperfusion-induced pathological changes, neurological complications have become one of the major problems for surgeons to solve (Bartels et al., 2014; Cefarelli et al., 2017). Previous studies reported neurological dysfunction in 5.3–25% of patients after DHCA (Leshnower et al., 2010; Hu et al., 2014; Leshnower et al., 2015; Centofanti et al., 2016). In recent years, many studies have reported the effects of intraoperative cerebral perfusion strategies and temperature management for cerebral protection (Apostolakis and Shuhaiber, 2007; Fan et al., 2019); nevertheless, the mechanism of neurologic morbidities has not been fully elucidated, and further investigations are warranted.

Long noncoding RNAs (lncRNAs) are a class of RNAs whose length is greater than 200 nt, and they regulate a variety of biological processes at the transcriptional, posttranscriptional and epigenetic levels (Lalevée and Feil, 2015; Yu et al., 2018). Studies have reported that lncRNAs participate in multiple physiological functions, such as gene transcriptional regulation, chromatin modification, ontogenetic regulation, cell programming, and stem cell pluripotency maintenance in various ways (Clark et al., 2012). LncRNAs are specifically highly expressed in the central nervous system and regulate central nervous system development and neurodegenerative diseases (Ng et al., 2013). Studies have demonstrated that lncRNAs were differentially expressed in the ischemic brain after stroke, and that alteration to this expression was closely related with changes in gene expression, suggesting lncRNAs may regulate brain injury by altering the expressions of associated genes (Zhang et al., 2016; Liu et al., 2018). Feng et al. found that the expression of lncRNA ANRIL in patients with acute ischemic stroke (AIS) was lower than that in normal people, suggesting that ANRIL was closely related to the degree of cerebral inflammation after stroke (Feng et al., 2019). However, very few studies have reported the role of lncRNAs in DHCA.

MiRNAs are a class of ubiquitous, conserved, and endogenous noncoding single-stranded RNAs that are usually 19–25 nucleotides in length, and they specifically recognize and target mRNAs to promote their degradation and act as regulatory factors in the posttranscriptional stage of genes (Filipowicz et al., 2008; Zhang et al., 2019). An increasing number of noncoding RNAs have been found to be involved in physiological and pathological processes of the nervous system (Zhong et al., 2019). LncRNAs can serve as competitive endogenous RNAs (ceRNAs) to specifically bind with miRNAs, thus reducing mRNA degradation (Wang C. et al., 2020). For example, Gao’s group found that after DHCA, the lncRNA GAS5 was significantly increased in the hippocampus and could directly bind to miR-23a to promote the expression of the downstream PTEN gene. PTEN can decrease the levels of p-Akt and Bcl-2 through the dephosphorylation of PIP3, thereby inducing neuronal apoptosis (Gao et al., 2019). Ma et al. presented lncRNA-associated ceRNA profiles in an Alzheimer’s disease (AD) mouse brain and identified lncRNA-associated ceRNA networks (Ma et al., 2020). However, the roles and mechanisms of lncRNAs and the analysis of the lncRNA-associated ceRNA network in DHCA have not been comprehensively explored.

Transcription factors (TFs) are DNA-binding proteins that can play significant roles in the regulation of gene expression (Redell and Tweardy, 2006). MiRNA-TF coregulation is one of the most important feed-forward loop (FFL) types and represents a powerful method for investigating the underlying global relationships between molecular networks (Cai et al., 2021). Many studies have revealed that TFs regulate gene expression by interacting with miRNAs, for example, Mangalhara et al. revealed the zinc finger E-box binding homeobox 1(ZEB1) can downregulate miR-101 expression by regulating miR-101-1 promoter and induce Epithelial-Mesenchymal Transition (EMT) in breast cancer (Chandra Mangalhara et al., 2017). Understanding the interaction between these two regulators and their targets is crucial for revealing the complex molecular regulatory mechanisms of DHCA cerebral injury; however, detailed reports are not currently available on the TFs and TF-miRNA-mRNA network in DHCA.

**FIGURE 1:**
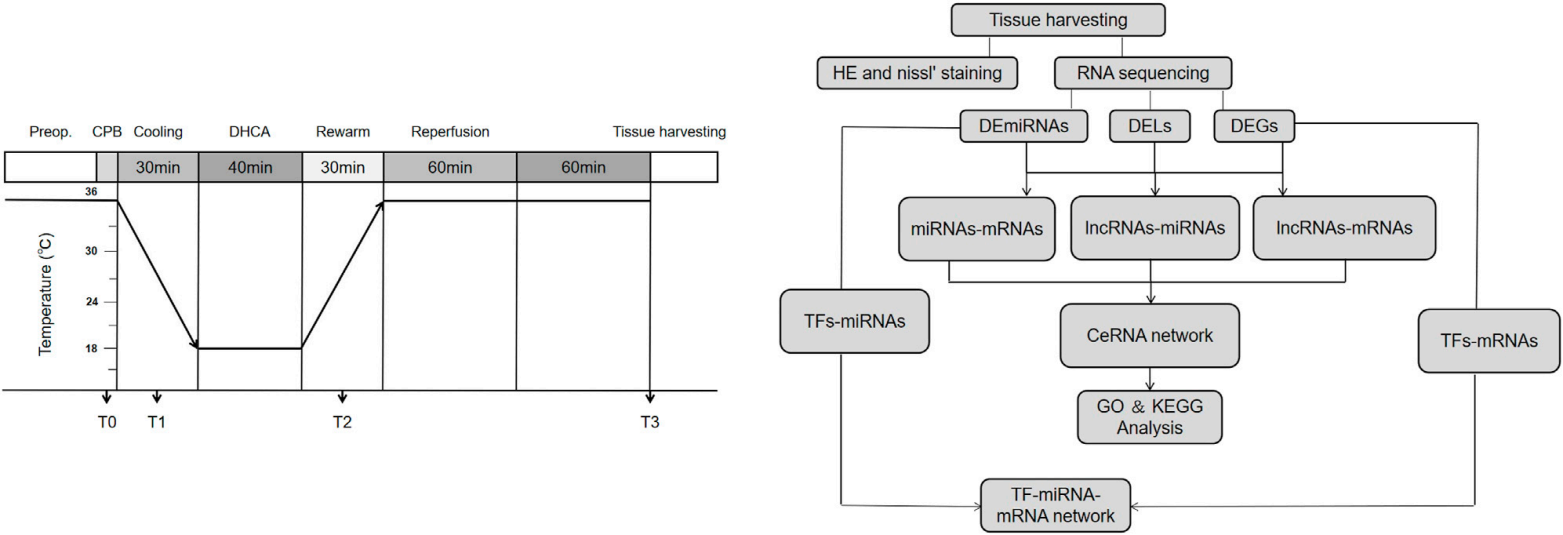
The workflow and experimental protocol. Preop, Pre-operation; CPB, cardiopulmonary bypass; DHCA, deep hypothermic circulatory arrest.

In this study, we investigated the differentially expressed profiles of lncRNAs, miRNAs and mRNAs in the brain cortex of DHCA rats through high-throughput sequencing, quantitative real-time PCR (qRT-PCR) verification and bioinformatics analysis. Then, we analyzed the GO and KEGG pathways of DERNAs and established lncRNA-miRNA-mRNA and TF-miRNA-mRNA coregulatory networks. This study aims to provide insights into the mechanism of brain injury after DHCA and guidance for further steps in treatment strategies. The workflow chart and schematic diagram of the present study were showed in Figure 1.

## 2 Materials and Methods

### 2.1 Animal Model of Deep Hypothermic Circulatory Arrest

All procedures performed on the animals were approved by the Institutional Animal Care Committee of Sun Yat-sen University and conducted in compliance with Guidelines for Animal Experimentation of Sun Yat-sen University (Approval No. SYSU-IACUC-2021-000181). Twenty age-matched male Sprague–Dawley rats (age, 12–16 weeks; weight, 350–450 g) were provided by the Sun-yat Sen University Animal Center and randomly allocated into a DHCA group and a sham group, as previously described (Tu et al., 2019). In short, the rats were anesthetized with pentobarbital sodium (intraperitoneal) and orotracheally intubated with a 14G cannula, ventilated with a tidal volume of 10 ml/kg and a respiratory rate of 60–65 beats min^−1^ and given systemic heparinization (200 IU). Then, the left and right carotid arteries were cannulated and a multistaged venous return cannula was placed next to the junction of the inferior vena cava and right atrium through the right external jugular vein. The CPB circuit included a venous reservoir, a peristaltic pump and a custom-designed small-volume oxygenator. CPB was instituted at a flow rate of 80–100 ml kg^−1^ min^−1^, and the flow rate was decreased during the 30-min cooling process. By using a heat exchanger, a cooling blanket and ice bags, rats were cooled to a rectal temperature of 18°C within 30 min. Thereafter, the CPB was stopped and venous blood was drained into the reservoir. The animals were subjected to DHCA for another 30 min, which was confirmed by electrocardiography. Forty minutes later, the CPB was restarted. The rats were rewarmed to a rectal temperature of 34°C over 40 min, and then CPB was discontinued. Then, these rats were ventilated for an additional 60 min and gradually rewarmed to a rectal temperature of 36–37°C. Rats in the sham group only received anesthesia, cannulation, and heparin management. Sixty min later, the rats were euthanized and the brain tissue was harvested immediately for high-throughput sequencing analysis. Information on body temperature, blood pressure, heart rate, pH and PCO_2_ value was collected at 4 time points: just before CPB (T0), after cooling for 20 min (T1), after rewarming for 20 min (T2) and at the end of DHCA (T3).

### 2.2 Hematoxylin-Eosin and Nissl’s Staining

Rats (three for each group) were anesthetized and sacrificed by cervical dislocation, and the brain tissue was immediately extracted and placed on ice. The brain tissue was fixed overnight with 4% paraformaldehyde, embedded in paraffin, and sliced into serial sections with a thickness of 3–4 μm consecutively. For HE staining, the sections were deparaffinized, debenzolized, rinsed with water, and stained with hematoxylin for 8 min. The sections were then washed in running water for 5 min and stained with eosin for 2 min. For Nissl’s staining, the tissues were dewaxed to distilled water, stained with 1% toluidine blue solution at 60°C for 40 min, washed with distilled water for 3 min, then decolorized in gradient alcohol for about 3 min. Finally, sections were routinely dehydrated, cleared and resin-sealed. The morphological changes of cells were observed under a light microscope and the neuronal Nissl bodies were stained blue and purple.

### 2.3 RNA Isolation

Total RNA was extracted by Trizol reagent (Invitrogen, CA, United States), RNA degradation and contamination were monitored on 1% agarose gels, RNA purity was checked using the NanoPhotometer® spectrophotometer (IMPLEN, CA, United States), RNA concentration was measured using Qubit® RNA Assay Kit in Qubit® 2.0 Flurometer (Life Technologies, CA, United States), and RNA integrity was assessed using the RNA Nano 6000 Assay Kit of the Agilent Bioanalyzer 2100 system (Agilent Technologies, CA, United States).

### 2.4 Library Preparation, Clustering and Sequencing for RNA

A total amount of 3 μg total RNA per sample was used as input material for the small RNA library. Sequencing libraries were generated using NEBNext® Multiplex Small RNA Library Prep Set for Illumina® (NEB, United States of America) following manufacturer’s recommendations and index codes were added to attribute sequences to each sample. At last, library quality was assessed on the Agilent Bioanalyzer 2100 system using DNA High Sensitivity Chips. The clustering of the index-coded samples was performed on a cBot Cluster Generation System using TruSeq SR Cluster Kit v3-cBot-HS (Illumia) according to the manufacturer’s instructions. After cluster generation, the library preparations were sequenced on an Illumina Hiseq 2500/2000 platform and 50 bp single-end reads were generated. A total amount of 3 µg RNA per sample was used as input material for the RNA sample preparations. Sequencing libraries were generated using NEBNext® UltraTM RNA Library Prep Kit for Illumina® (NEB, United States of America) following manufacturer’s recommendations and index codes were added to attribute sequences to each sample. At last, PCR products were purified (AMPure XP system) and library quality was assessed on the Agilent Bioanalyzer 2100 system. The clustering of the index-coded samples was performed on a cBot Cluster Generation System using TruSeq PE Cluster Kit v3-cBot-HS (Illumia) according to the manufacturer’s instructions. After cluster generation, the library preparations were sequenced on an Illumina Hiseq platform and 125/150 bp paired-end reads were generated.

### 2.5 Quality Control and Quantification of Gene Expression Level

Raw data of fastq format were firstly processed through custom perl and python scripts. In this step, for small RNA, clean data were obtained by removing reads containing ploy-N, with 5′ adapter contaminants, without 3’ adapter or the insert tag, containing ploy A or T or G or C and low-quality reads from raw data. For Transcriptome sequencing, clean data were obtained by removing reads containing adapter, reads containing ploy-N and low-quality reads from raw data. At the same time, Q20, Q30, and GC content of the clean data were calculated. The small RNA tags were mapped to reference sequence by Bowtie without mismatch to analyze their expression and distribution on the reference (Langmead et al., 2009). MiRNA expression levels were estimated by TPM (transcript per million) through the following criteria: Normalization formula: Normalized expression = mapped readcount/Total reads*1000000. For Transcriptome sequencing, Reference genome and gene model annotation files were downloaded from genome website directly. Index of the reference genome was built using STAR and paired-end clean reads were aligned to the reference genome using STAR (v2.5.1b). HTSeq v0.6.0 was used to count the reads numbers mapped to each gene. And then FPKM of each gene was calculated based on the length of the gene and reads count mapped to this gene. The row data has been successfully deposited in GenBank, and the accession number is PRJNA739516.

### 2.6 Data Analysis

A DERNA analysis of the high-throughput sequencing data was performed using the edgeR R package (3.12.1) (Robinson et al., 2010). The resulting *p*-value were adjusted using the Benjamini and Hochberg’s approach for controlling the false discovery rate (FDR). The cutoff values for differentially expressed lncRNAs (DELs) and mRNAs (DEGs) were |log_2_ Fold Change| ≥2 and FDR<0.05, and for miRNAs (DEmiRNAs), |log_2_ Fold Change| ≥1.5 and *p* < 0.05. Volcano plots of DERNAs were generated using the ggplots2 R package, and a hierarchical clustering analysis was conducted using the pheatmap R package.

### 2.7 Construction the CeRNA Network

The DEL-associated ceRNA networks were constructed based on the competing endogenous RNA (ceRNA) hypothesis. The target miRNAs of lncRNAs were predicted based on the results of using miRanda (Enright et al., 2003), and the lncRNAs and their paired miRNAs exhibiting negatively correlated expressions were considered as lncRNA-miRNA pairs. By coexpression theory, the expressive correlation between DELs and DEGs was evaluated using the Pearson correlation coefficient (PCC), pairs with PCC > 0.95 were selected as lncRNA-mRNA pairs. The TargetScan (Agarwal et al., 2015), miRanda (Enright et al., 2003), and RNAhybrid (Rehmsmeier et al., 2004) databases were used to predict the biological targets of DEmiRNAs, the overlapping results between the targets of DEmiRNAs and DEGs were selected as miRNA-mRNA pairs. The ceRNA networks were visualized with DELs, DEmiRNAs and DEGs by using Cytoscape (version 3.8.2).

### 2.8 GO and KEGG Enrichment Analysis

GO and KEGG functional and pathway enrichment analyses of differentially expressed lncRNAs were implemented by the clusterProfiler R package (Yu et al., 2012), in which the gene length bias was corrected base on Wallenius non-central hyper-geometric distribution. Enriched biological process (BP), cellular component (CC), molecular function (MF) categories and KEGG pathways were used to analyze the DELs. A *p*-value < 0.05 was defined as statistically significant.

### 2.9 Establishing the TF-miRNA-mRNA and TF-lncRNA-mRNA Network

TransmiR v2.0 (http://www.cuilab.cn/transmir) (Tong et al., 2019) dataset was used to analyze the TF-miRNA, and ChIP-Atlas (http://chip-atlas.org/) (Oki et al., 2018) was used to analyze the TF-lncRNA and TF-mRNA, the threshold for statistical significance values calculated by peak-caller MACS2 was 50, and the distance range from the Transcription Start Sites (TSS) was between -1000 and +1000 bp. According to the above findings, the overlapping results were selected to established TF-miRNA-mRNA and TF-lncRNA-mRNA regulatory networks using Cytoscape (version 3.8.2) software to visualize their interactions.

### 2.10 Quantitative Real-Time Polymerase Chain Reaction

Total RNA was isolated using TRIzol reagent (Invitrogen, CA, United States). For lncRNAs and mRNAs, the extracted RNA was reverse transcribed into cDNA using Color Reverse Transcription kit (EZBioscience, Roseville, United States), and for miRNAs, the extracted RNA was reverse transcribed into cDNA using microRNA Reverse Transcription kit (EZBioscience, Roseville, United States) according to the manufacturer’s instructions. Quantitative real-time polymerase chain reaction (qRT-PCR) was performed to select mRNAs (Ttr, Tfpi2, Ccl2, Lhx5 and Cxcl1) and lncRNAs (Rph3a, Smim13, AABR07066379.1, LINC2085 and Lcorl) using SYBR Green qPCR Master Mix (EZBioscience, Roseville, United States) and to select miRNAs (miR-450b-3p, miR-200b-3p, miR-200a-3p and miR-429) using EZ-Probe qPCR Master Mix for microRNA (EZBioscience, Roseville, United States) according to the manufacturer’s instructions. Relative expression levels were normalized to GAPDH and U6 as internal standard controls for selected DERNAs, and they were calculated by the 2^−ΔΔCt^ method. Specific primers were designed as shown in Table 1.

**TABLE 1:** Primers for the quantitative real-time PCR (qRT-PCR) assay of selected DERNAs.

Gene names	Category	Primer sequence (5′-3′)
Rph3a	lncRNA	F: CAC​AAT​GCA​TCG​CGG​TGG​G
		R: CTT​CCA​CTT​CGT​GGC​ACT​CG
Smim13	lncRNA	F: GACCTCGCGTGAGTCCG
		R: CGG​ACG​TCG​AGG​GAG​AAA​G
AABR07066379.1	lncRNA	F: CCA​CTG​TGT​ATG​CTT​GCT​GTC
		R: CCC​GAT​TGC​TTT​AAA​CTC​CCT
LINC2085	lncRNA	F: TGG​TGG​GTG​AAA​ACA​GAG​GC
		R: GCA​GAT​GAC​ATT​TTC​TAG​GCA​GT
Lcorl	lncRNA	F: ACT​GTG​ACT​CGA​ACG​CAT​GA
		R: AGC​AGT​CTC​CGT​TCT​CCA​TTC
miR-450b-3p	miRNA	F: ATT​GGG​GAC​GCT​TCG​CAT​TCA
miR-200b-3p	miRNA	F: GCG​GCT​AAT​ACT​GCC​TGG​TAA​TG
miR-200a-3p	miRNA	F: GCG​GTA​ACA​CTG​TCT​GGT​AAC​G
miR-429	miRNA	F: CGG​CTA​ATA​CTG​TCT​GGT​AAT​GCC
miRNA		R: CTCAACTGGTGTCGTGGA
Ttr	mRNA	F: CGG​AAG​GGG​TGT​ACA​GGG​TA
		R: TGG​CTG​TGA​AAA​CCA​CCT​CT
Tfpi2	mRNA	F: CTT​CAG​TAT​CTG​CCC​AAG​GGA​AT
		R: ATT​TCC​GCA​AGT​GTG​TTC​GC
Ccl2	mRNA	F: TGT​CTC​AGC​CAG​ATG​CAG​TT
		R: CAG​CCG​ACT​CAT​TGG​GAT​CA
Lhx5	mRNA	F: CGA​CTT​CTT​CAG​GCG​CTT​TG
		R: AGG​TGG​AAG​ACT​TTG​CTC​CG
Cxcl1	mRNA	F: CAC​ACT​CCA​ACA​GAG​CAC​CA
		R: GCG​GCA​TCA​CCT​TCA​AAC​TC
TNF-α	mRNA	F: ATC​GGT​CCC​AAC​AAG​GAG​GA
		R: GCT​TGG​TGG​TTT​GCT​ACG​A
TNFR	mRNA	F: CCA​AGT​GCC​ACA​AAG​GAA​CC
		R: GTG​CCT​TTA​TCA​CAC​ACC​TCG
NF-κB	mRNA	F: CAC​CGT​GAC​AGC​AGG​ACC​CAA​G
		R: GTA​GAT​AGG​CAA​GGT​CAG​AAT​G
IL-6	mRNA	F: CCC​ACC​AGG​AAC​GAA​AGT​CAA
		R: CTG​GAA​GTC​TCT​TGC​GGA​G
IL-1β	mRNA	F: GGC​TTC​CTT​GTG​CAA​GTG​TC
		R: AGT​CAA​GGG​CTT​GGA​AGC​AA
TLR1	mRNA	F: CTG​CTT​TGG​GGA​GCA​ACA​AC
		R: ACC​CAA​ATT​ATA​GTA​CCC​CGC
TLR2	mRNA	F: GGA​GGT​CTC​CAG​GTC​AAA​TC
		R: ACA​CAC​CAG​CAG​CAT​CAC​AT
GAPDH	mRNA	F: CGA​CTT​CTT​CAG​GCG​CTT​TG
		R: AGG​TGG​AAG​ACT​TTG​CTC​CG
U6		F: CTCGCTTCGGCAGCACA
		R: AAC​GCT​TCA​CGA​ATT​TGC​GT

### 2.11 Detection of Tumor Necrosis Factor-α and Western Blot

After 2 h of reperfusion, 5 rats in each group were killed by decapitation. The brain cortex of rat was harvested. Proteins were extracted from tissues in cold lysis buffer. Tumor necrosis factor (TNF)-α levels in brain cortex samples were detected using commercially available ELISA Kits (Elabscience, Wuhan, China) with the recommended protocol by the manufacturer. Protein expression levels of TNFR, NF-κB p65, IL-6, TLR1 and TLR2 were further determined with western blot analysis by using polyclonal antibodies against them (1:1000 dilution; Affinity Biosciences, United States). Western blotting was performed as standard protocol. Signals were detected with an enhanced chemiluminescence system (Millipore, Billerica, MA, United States). The protein expressions were analyzed with National Institute of Health (NIH) Image (Research Services Branch, National Institutes of Health, Bethesda, Maryland) and quantified as a relative fold to the control group after normalization with GAPDH.

### 2.12 Statistical Analysis

Statistical analysis was performed using SPSS (version 25; IBM, Armonk, NY, United States) and presented graphically using GraphPad Prism 8.0 (GraphPad, La Jolla, CA, United States). Numerical data are presented as the mean ± standard deviation. Differences between means were analyzed using Student’s t-test. A *p*-value less than 0.05 was considered statistically significant.

## 3 Results

### 3.1 Characteristics of the DHCA and Sham Rats

**FIGURE 2:**
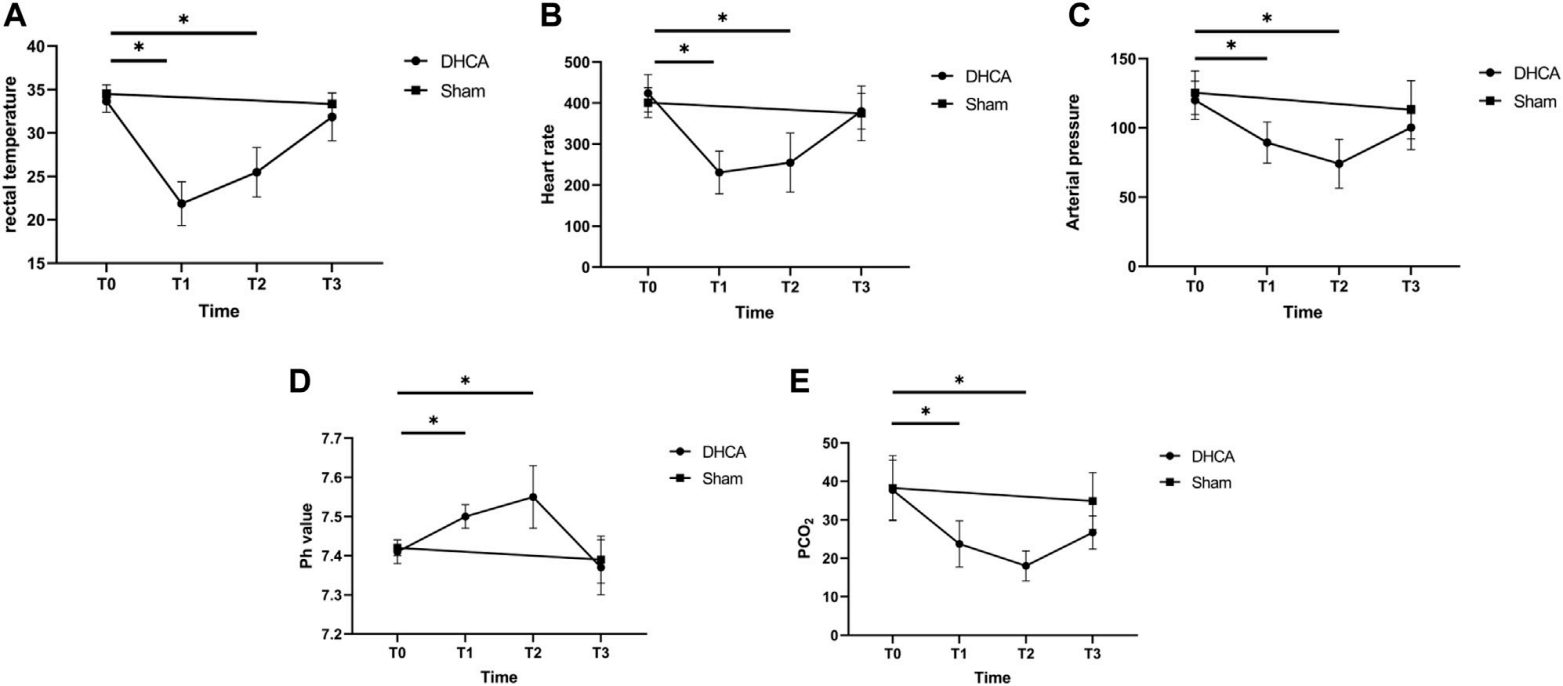
Characteristics of the DHCA and sham rats at four time points in DHCA. **(A)** Rectal temperature. **(B)** Heart rate. **(C)** Arterial pressure. **(D)** pH value. **(E)** PCO_2_. *n* = 5 per group. *p < 0.05 vs T0. Just before CPB (T0), after cooling down for 20 min (T1), after rewarming for 20 min (T2), and at the end of DHCA (T3).

**FIGURE 3:**
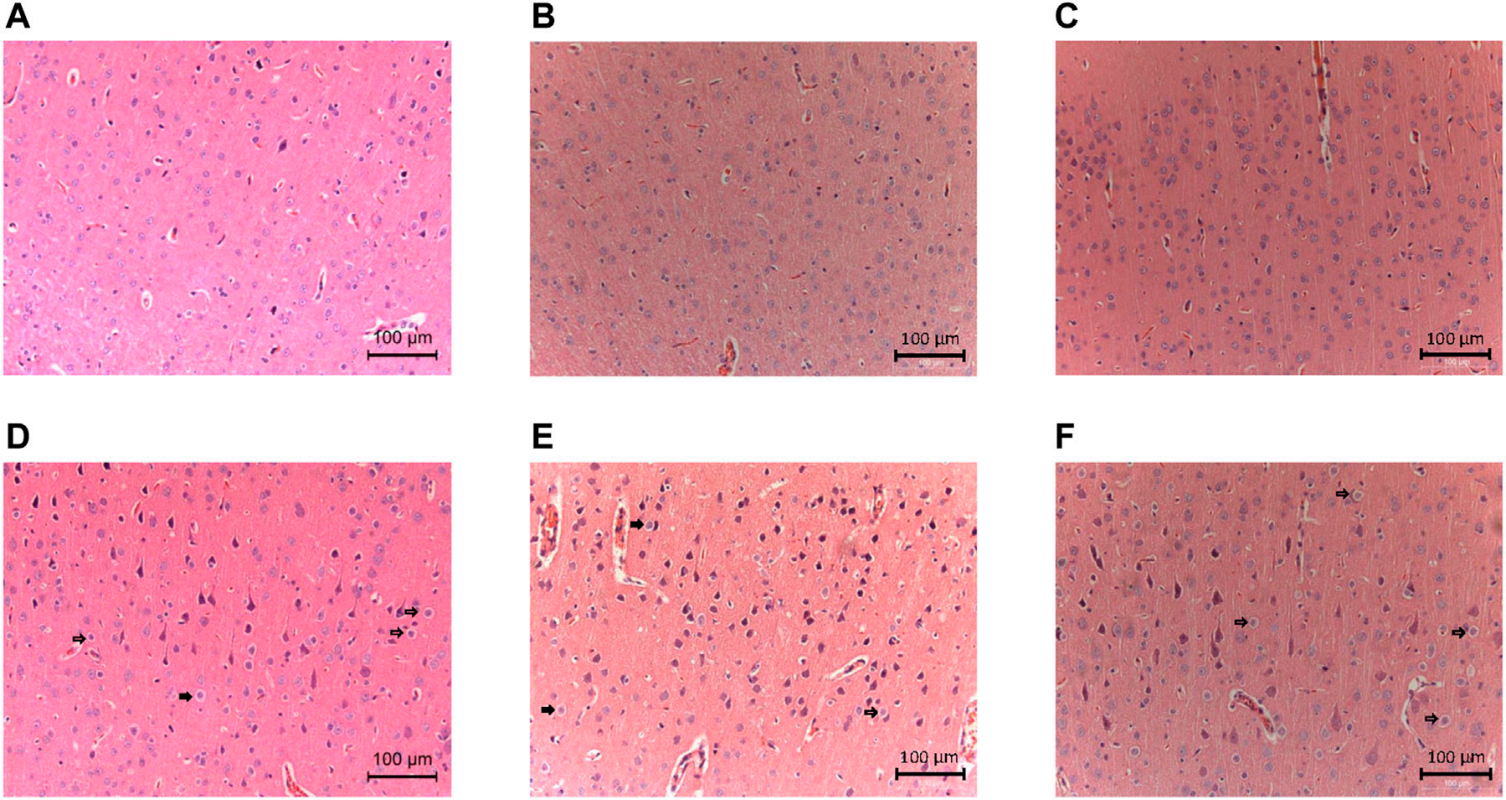
Neurologic damages in the cortex induced by DHCA (*n* = 3, each picture represents a rat). **(A–C)** Sham groups. The neurons were normal in shape with obvious nucleus and uniform mesenchyme. **(D–F)** DHCA groups. Obvious swelling, deformation and nuclear pyknosis of neurons (Indicated by the arrow).

**FIGURE 4:**
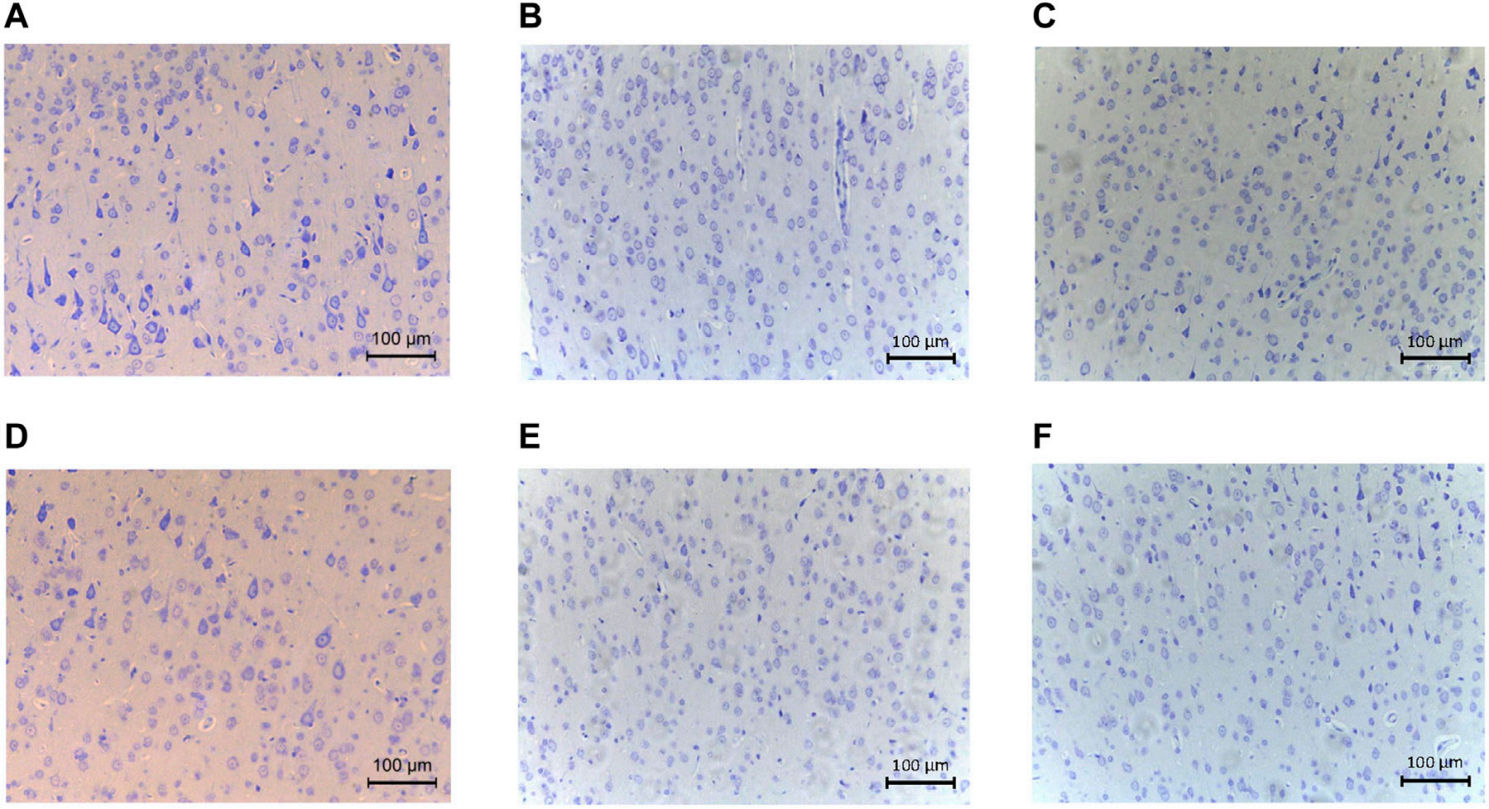
The Nissl’s staining showed pathological changes of cortex tissues (*n* = 3, each picture represents a rat). **(A–C)** Nissl’s body staining in cortex of sham group. **(D–F)** Nissl’s body staining in cortex of DHCA group.

Detailed characteristics of the DHCA and sham rats are shown in Figure 2. For validation, significant differences were observed in the rectal temperature (Figure 2A), heart rate (Figure 2B), arterial pressure (Figure 2C), pH (Figure 2D) and PCO_2_ (Figure 2E) value between the DHCA and sham groups (all *p* < 0.05). In the sham groups, the neurons in brain tissue were normal, the nucleus were obvious and the mesenchyme was uniform (Figures 3A–C). In the DHCA groups, obvious swelling, distortion, nuclear pyknosis and nuclear disappearance were observed (Figures 3D–F). For Nissl’s staining, the sham group showed that neuronal Nissl bodies were stained blue (Figures 4A–C), however the number of blue Nissl body decreased significantly in the DHCA group (Figures 4D–F).

### 3.2 Differential Expression of DERNAs in the DHCA and Sham Groups

**FIGURE 5:**
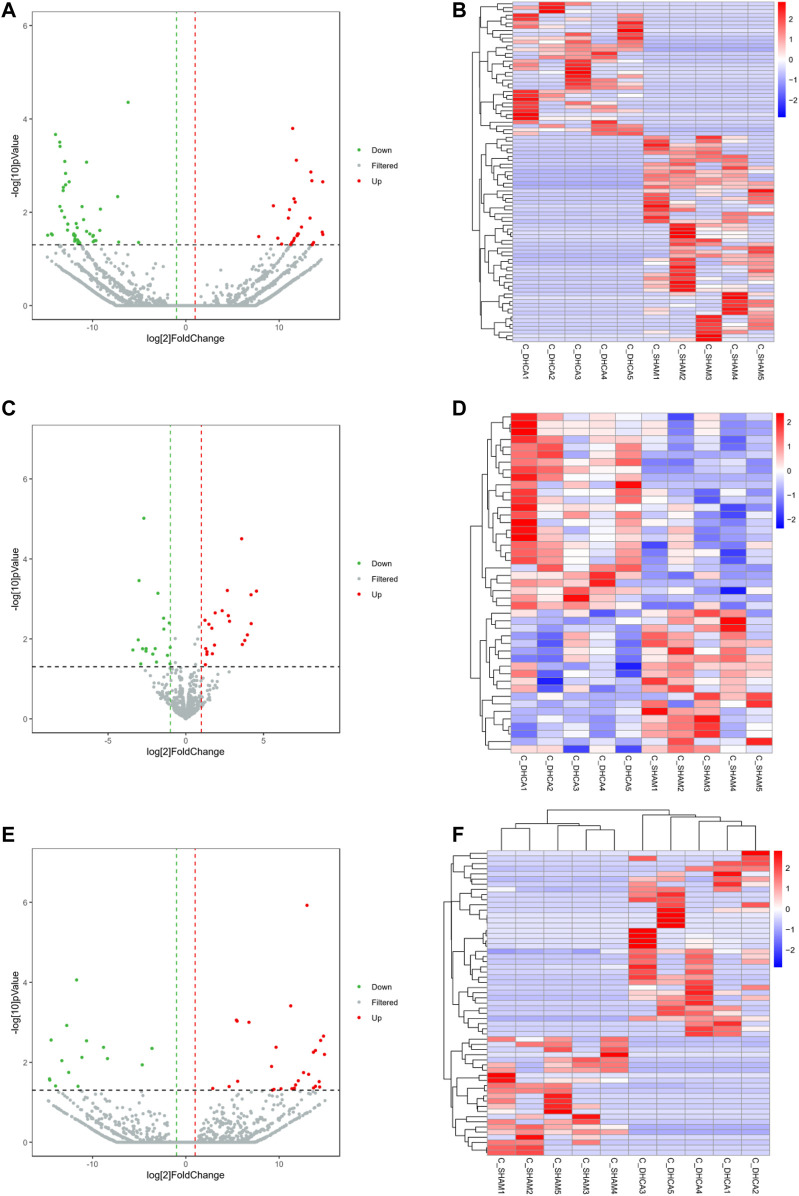
Differentially expressed RNAs between DHCA and sham rats. Volcano plots and heat map of the lncRNA **(A,B)**, miRNA **(C,D)** and mRNA **(E,F)** expression profiles between the DHCA and sham groups. The vertical lines represented a *p*-value of 0.05 in log10 scale, the horizontal lines represented up and down regulation with 2 fold-change. The red points represented the upregulated genes, the green points represented the downregulated genes, and the gray points represented genes that were not significantly differentially expressed.

High-throughput sequencing analysis was conducted to detect differential lncRNA, miRNA and mRNA expression between the DHCA and sham groups. All the DELs, DEmiRNAs and DEGs are presented by hierarchical clustering heat maps and volcano plots in Figure 5. After data normalization, a total of 89 DELs were revealed, and they included 35 upregulated and 54 downregulated lncRNAs (Figures 5A,B). Twenty-five miRNAs were found to be significantly increased, and 20 miRNAs were significantly decreased (Figures 5C,D). Among the DEGs, thirty-six were upregulated and 23 were downregulated in the DHCA group compared with the sham group (Figures 5E,F). The results of DERNAs were shown in Tables 2–4.

**TABLE 2:** The RNA-seq result of the differentially expressive mRNAs.

Gene name	Regulate	Log2(Fold change)	*p* Value	FDR
Fyn	Up	18.08257	5.31E-06	0.004446
Gria1	Up	17.19211	0.000201	0.044503
XLOC_163769	Up	17.18823	8.23E-06	0.005595
Slc4a5	Up	17.0262	5.16E-07	0.000885
Lrba	Up	16.68526	0.000222	0.047552
Ppard	Up	15.45752	3.05E-05	0.014001
Mta1	Up	15.16327	8.00E-07	0.001201
Rps27a	Up	15.00067	1.23E-06	0.001643
Sytl2	Up	14.8686	1.02E-05	0.00635
Akap2	Up	14.76709	1.84E-06	0.002225
XLOC_056290	Up	14.45676	2.64E-06	0.002835
Cltrn	Up	14.35137	0.000172	0.041565
Kdm5b	Up	14.29894	0.0001	0.030499
Hmcn1	Up	13.90118	6.85E-06	0.005055
Rrn3	Up	13.90041	0.000159	0.039954
Ngf	Up	13.69297	0.000193	0.043957
Irf2	Up	13.68096	8.31E-06	0.005599
XLOC_050473	Up	13.16835	5.31E-05	0.020034
Calr4	Up	12.99354	8.25E-11	1.19E-06
Lrig1	Up	12.62189	4.58E-05	0.018129
XLOC_093586	Up	12.05918	9.07E-05	0.029026
XLOC_195631	Up	11.76973	0.000138	0.03694
Lgals3	Up	11.64316	0.000201	0.044503
Spata17	Up	11.37483	0.000209	0.04576
Arhgef10l	Up	11.24498	1.48E-07	0.000388
Rcc1	Up	10.20031	0.000212	0.04605
Ttr	Up	9.67349	4.89E-06	0.004197
Capn13	Up	9.4445	0.000223	0.047644
Svopl	Up	9.281385	0.00024	0.049878
Psd3	Up	9.189205	2.63E-05	0.012726
Ccl2	Up	6.962498	0.000134	0.048532
Kl	Up	5.563768	9.64E-05	0.029917
Cyp7b1	Up	5.503412	5.59E-07	0.000936
Tmem72	Up	5.494423	2.86E-05	0.019587
Cxcl1	Up	4.649312	0.000165	0.04052
Epb41l3	Up	2.889026	0.000205	0.045136
Gabrg3	Down	−3.6265	5.46E-06	0.004489
Grm3	Down	−4.66756	2.28E-05	0.011587
COX3	Down	−8.39863	1.43E-05	0.00809
S100a3	Down	−8.82519	4.98E-06	0.004223
Flot2	Down	−10.6485	2.77E-06	0.002894
Zfp92	Down	−11.1431	1.30E-05	0.007532
XLOC_400923	Down	−11.5614	0.00016	0.039954
Calr4	Down	−11.7122	1.45E-08	8.70E-05
Plod2	Down	−12.5617	4.50E-05	0.017948
Npnt	Down	−12.7704	7.96E-07	0.001201
Vps29	Down	−13.2978	1.70E-05	0.009107
XLOC_070906	Down	−13.9725	0.000152	0.039186
LOC108348298	Down	−14.4482	2.54E-06	0.002774
Ano2	Down	−14.5858	8.37E-05	0.027929
RGD1309730	Down	−14.6005	8.21E-05	0.027631
LOC103693999	Down	−14.6226	7.66E-05	0.026041
Dclk2	Down	−15.0215	7.84E-10	7.06E-06
Hectd2	Down	−15.1719	3.67E-06	0.003433
XLOC_070906	Down	−15.2193	4.07E-05	0.01699
Rpl23a	Down	−15.7447	2.20E-05	0.0114
Sncaip	Down	−15.8849	2.21E-07	0.000549
Srp68	Down	−17.2822	0.000178	0.042
Arid1b	Down	−18.9137	0.000117	0.032742

**TABLE 3:** The RNA-seq results of the differentially expressive miRNAs.

Gene name	Regulate	Log2(Fold change)	*p* Value	FDR
novel_623	Up	4.5483	0.00064	0.083127
novel_576	Up	4.2088	0.004149	0.19793
novel_470	Up	4.1984	0.000791	0.083127
novel_797	Up	3.964	0.007945	0.27844
novel_365	Up	3.7961	0.010943	0.35018
rno-miR-632	Up	3.6375	0.01382	0.98432
rno-miR-155-3p	Up	3.5941	3.14E-05	0.099145
rno-miR-1912-3p	Up	2.8138	0.00362	0.19793
novel_249	Up	2.7376	0.002629	0.19353
rno-miR-1298	Up	2.673	0.000617	0.083127
rno-miR-448-3p	Up	2.3388	0.001983	0.18247
rno-miR-34b-5p	Up	1.8899	0.002249	0.18388
rno-miR-218a-1-3p	Up	1.8455	0.014315	0.40522
rno-miR-141-5p	Up	1.7088	0.023591	0.47169
rno-miR-34c-3p	Up	1.6744	0.005412	0.22039
rno-miR-34c-5p	Up	1.4852	0.004303	0.19793
rno-miR-34b-3p	Up	1.3627	0.020969	0.45391
rno-miR-450b-3p	Up	1.3554	0.024354	0.47169
novel_202	Up	1.2799	0.017525	0.41947
rno-miR-3473	Up	1.2589	0.044358	0.96479
rno-miR-204-5p	Up	1.2322	0.003416	0.96479
rno-miR-27a-5p	Up	0.85824	0.005022	0.27844
rno-miR-130b-5p	Up	0.76052	0.010059	0.47169
rno-miR-92b-5p	Up	0.62565	0.043752	0.91706
rno-miR-1843b-3p	Up	0.58205	0.013582	0.68822
rno-miR-451-5p	Down	-0.67498	0.039221	0.4763
rno-miR-146a-5p	Down	-0.68251	0.009812	0.61836
rno-miR-214-3p	Down	-1.0063	0.016369	0.41947
rno-miR-217-5p	Down	-1.0205	0.042135	0.96479
rno-miR-144-3p	Down	-1.0672	0.004005	0.19793
rno-miR-483-3p	Down	-1.183	0.025886	0.4763
rno-miR-3548	Down	-1.4168	0.00569	0.22039
rno-miR-200a-5p	Down	-1.4322	0.003036	0.19793
rno-miR-200b-3p	Down	-1.7973	0.000725	0.083127
rno-miR-216a-5p	Down	-1.889	0.038114	0.96479
novel_267	Down	-1.9735	0.017557	0.41947
rno-miR-133a-5p	Down	-2.1275	0.024019	0.47169
novel_360	Down	-2.5493	0.017595	0.41947
rno-miR-20b-3p	Down	-2.5605	0.020309	0.45295
rno-miR-200a-3p	Down	-2.6961	9.62E-06	0.00708
rno-miR-10a-3p	Down	-2.7784	0.017668	0.41947
novel_583	Down	-2.8941	0.04224	0.98432
rno-miR-429	Down	-3.0061	0.000348	0.083127
rno-miR-210-5p	Down	-3.0571	0.010585	0.35018
novel_549	Down	-3.4027	0.018999	0.43697

**TABLE 4:** The RNA-seq results of the differentially expressive lncRNAs.

Gene name	Regulate	log2(Fold change)	*p* Value	FDR
AABR07000398.1	Up	18.72582	0.000118	0.032742
AABR07015078.1	Up	17.19116	0.000186	0.042901
XLOC_031671	Up	16.45423	1.24E-05	0.007279
AABR07000398.1	Up	16.06837	2.32E-07	0.000557
XLOC_180167	Up	15.83574	2.28E-05	0.011587
AABR07066379.1	Up	15.48974	3.07E-05	0.014001
XLOC_183760	Up	15.03829	9.58E-07	0.00137
XLOC_114219	Up	14.69478	9.67E-05	0.029917
AC132752.1	Up	14.68351	1.85E-06	0.002225
XLOC_032047	Up	14.62858	7.89E-05	0.026679
AABR07000740.2	Up	13.69784	0.000201	0.044503
XLOC_401709	Up	13.58447	0.000236	0.049338
XLOC_296780	Up	13.54477	0.000235	0.049338
XLOC_032047	Up	13.51465	1.61E-06	0.002109
XLOC_233713	Up	13.40847	9.70E-07	0.00137
XLOC_321073	Up	13.32126	2.87E-05	0.01339
XLOC_183260	Up	12.40403	5.61E-05	0.020714
AABR07028665.1	Up	12.06398	9.04E-05	0.029026
XLOC_183260	Up	11.99925	8.93E-05	0.029026
XLOC_001184	Up	11.95685	0.000109	0.031403
XLOC_130708	Up	11.91075	0.00011	0.031785
Lcorl	Up	11.8507	3.75E-07	0.000769
XLOC_002265	Up	11.72053	9.43E-06	0.006065
XLOC_178536	Up	11.62521	0.000134	0.036176
XLOC_199402	Up	11.59374	7.12E-06	0.005138
XLOC_383778	Up	11.54971	0.000163	0.040346
XLOC_353926	Up	11.45387	3.32E-08	0.00016
XLOC_016969	Up	11.37004	0.000197	0.04441
XLOC_251786	Up	11.24147	0.000231	0.049013
XLOC_146802	Up	11.12149	1.61E-05	0.008811
XLOC_423414	Up	10.99514	2.88E-05	0.01339
XLOC_185405	Up	10.2686	0.000226	0.048019
XLOC_397006	Up	9.860291	0.000133	0.035938
XLOC_248812	Up	9.385525	1.24E-05	0.007279
LOC100366054	Up	7.823484	0.00012	0.033224
XLOC_054282	Down	−5.05733	0.000196	0.044374
AABR07066379.1	Down	−6.18503	6.75E-09	4.42E-05
XLOC_309763	Down	−7.19536	0.00019	0.043581
Rph3a	Down	−7.30309	5.72E-06	0.004628
XLOC_392693	Down	−9.15417	1.55E-05	0.008589
Tigd2	Down	−9.21973	6.95E-05	0.024418
XLOC_091451	Down	−9.64018	0.000164	0.040488
XLOC_163827	Down	−9.79889	0.000167	0.040702
XLOC_117584	Down	−9.9149	0.000108	0.031403
XLOC_012474	Down	−9.92959	0.000185	0.042815
XLOC_011788	Down	−9.96965	0.000115	0.03271
XLOC_184484	Down	−10.2947	9.15E-05	0.029026
XLOC_401380	Down	−10.6423	7.33E-05	0.025644
XLOC_051334	Down	−10.6437	4.47E-07	0.000847
XLOC_032116	Down	−10.9615	3.32E-05	0.014431
XLOC_414249	Down	−11.1294	1.32E-05	0.007574
XLOC_031619	Down	−11.3765	0.000205	0.045136
XLOC_163636	Down	−11.5196	0.000176	0.04194
XLOC_027988	Down	−11.5655	0.0002	0.044503
XLOC_400082	Down	−11.5768	0.000219	0.047191
XLOC_225246	Down	−11.6161	0.000151	0.039042
XLOC_345239	Down	−11.6469	5.87E-05	0.021356
14-Sep	Down	−11.6702	0.000151	0.039045
XLOC_356660	Down	−11.7588	4.27E-05	0.017492
Cdk7	Down	−11.87	0.000107	0.031321
XLOC_315310	Down	−11.8905	0.000116	0.032742
XLOC_230702	Down	−11.8968	0.000144	0.038018
XLOC_418655	Down	−11.9005	0.000104	0.030937
XLOC_178559	Down	−11.9195	0.000174	0.041565
XLOC_306130	Down	−12.0121	9.14E-05	0.029026
XLOC_148477	Down	−12.0383	0.000103	0.030693
XLOC_155181	Down	−12.5111	1.83E-06	0.002225
LOC100361934	Down	−12.657	4.46E-05	0.017948
XLOC_205095	Down	−12.6882	6.79E-05	0.024107
LOC363337	Down	−12.8944	2.33E-06	0.002579
AABR07056156.1	Down	−12.8968	1.08E-06	0.001469
XLOC_093120	Down	−12.9797	4.19E-07	0.000817
Olr901	Down	−13.0074	2.71E-05	0.012843
XLOC_031773	Down	−13.1341	2.88E-06	0.002924
XLOC_053865	Down	−13.1538	3.56E-06	0.003433
LOC100361645	Down	−13.3162	1.78E-05	0.009331
AABR07026893.1	Down	−13.488	1.41E-07	0.000388
XLOC_366938	Down	−13.5056	1.31E-05	0.007532
XLOC_381812	Down	−13.5229	9.61E-08	0.000315
XLOC_401383	Down	−13.975	5.38E-08	0.000215
XLOC_381812	Down	−14.3208	0.000101	0.030499
RGD1560108	Down	−14.4169	8.94E-05	0.029026
XLOC_157613	Down	−14.8173	0.000105	0.031054
XLOC_114219	Down	−15.3507	3.32E-05	0.014431
Smim13	Down	−15.3795	5.02E-07	0.000883
XLOC_031671	Down	−15.5167	3.30E-05	0.014431
XLOC_262441	Down	−16.7355	9.37E-08	0.000315
LOC103690050	Down	−16.8079	9.25E-06	0.006057
XLOC_191721	Down	−17.2889	0.000177	0.042

### 3.3 Validation of the Candidate Differential Genes

**FIGURE 6:**
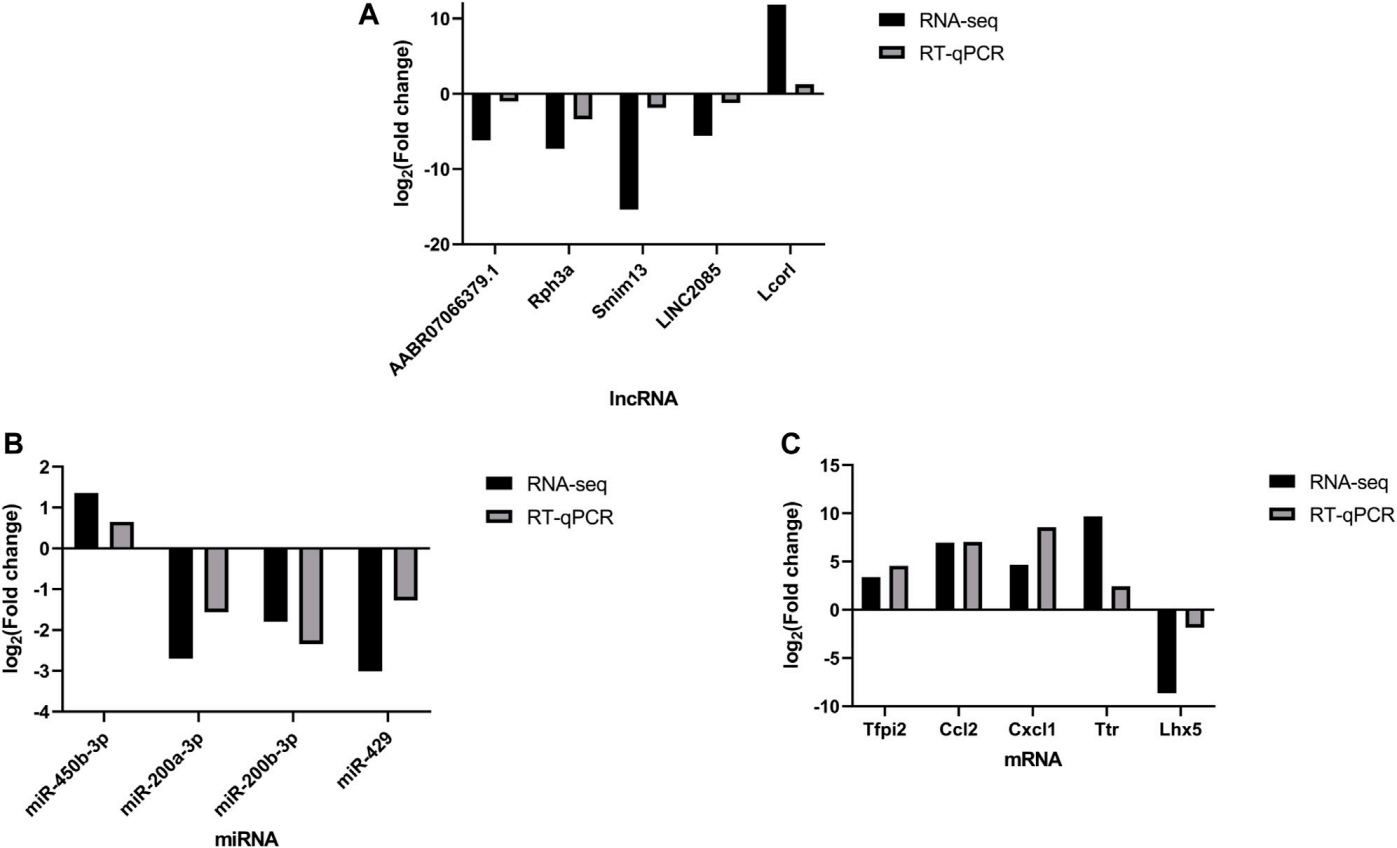
qPCR validation. Comparison between the RNA-seq and qPCR results of selected differentially expressed lncRNAs (AABR07066379.1, Rph3a, Smim13, LINC2085 and Lcorl) **(A)**, miRNAs (miR-450b-3p, miR-200b-3p, miR-200a-3p and miR-429) **(B)** and mRNAs (Tfpi2, Ccl2, Cxcl1, Ttr, and Lhx5) **(C)**. The qPCR analysis results were consistent with the RNA-seq data. All reactions were repeated three times for each mRNA and IncRNA, GAPDH and U6 were used as an internal control, *n* = 5.

The qRT-PCR was performed to verify the expression level changes gained from the RNA-seq analysis. We randomly selected 5 lncRNAs (Figure 6A), 4 miRNAs (Figure 6B) and 5 mRNAs (Figure 6C) with high fold changes to validate the RNA-seq results. These results were consistent with the high-throughput sequencing results and therefore were indicative of reliable high-throughput sequencing data. The results of fold change of these selected DERNAs were shown in Supplementary Figure S1.

### 3.4 GO Annotation and KEGG Pathway Analysis

**FIGURE 7:**
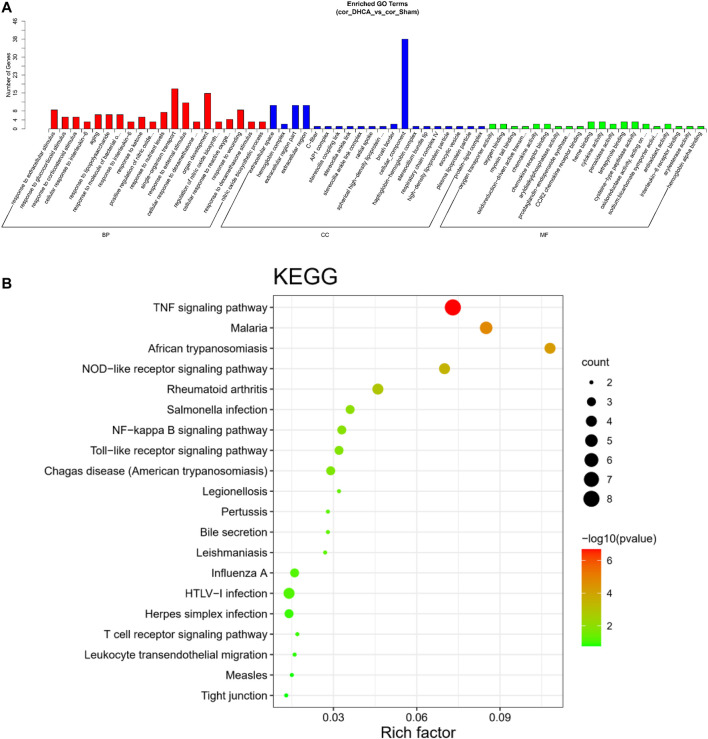
GO and KEGG pathway analysis of differentially expressed lncRNAs in DHCA. **(A)** Enriched lncRNAs were functionally classified according to the biological process (BP), cellular component (CC) and molecular function (MF) categories. **(B)** Top 20 significantly enriched KEGG pathway terms were shown.

**FIGURE 8:**
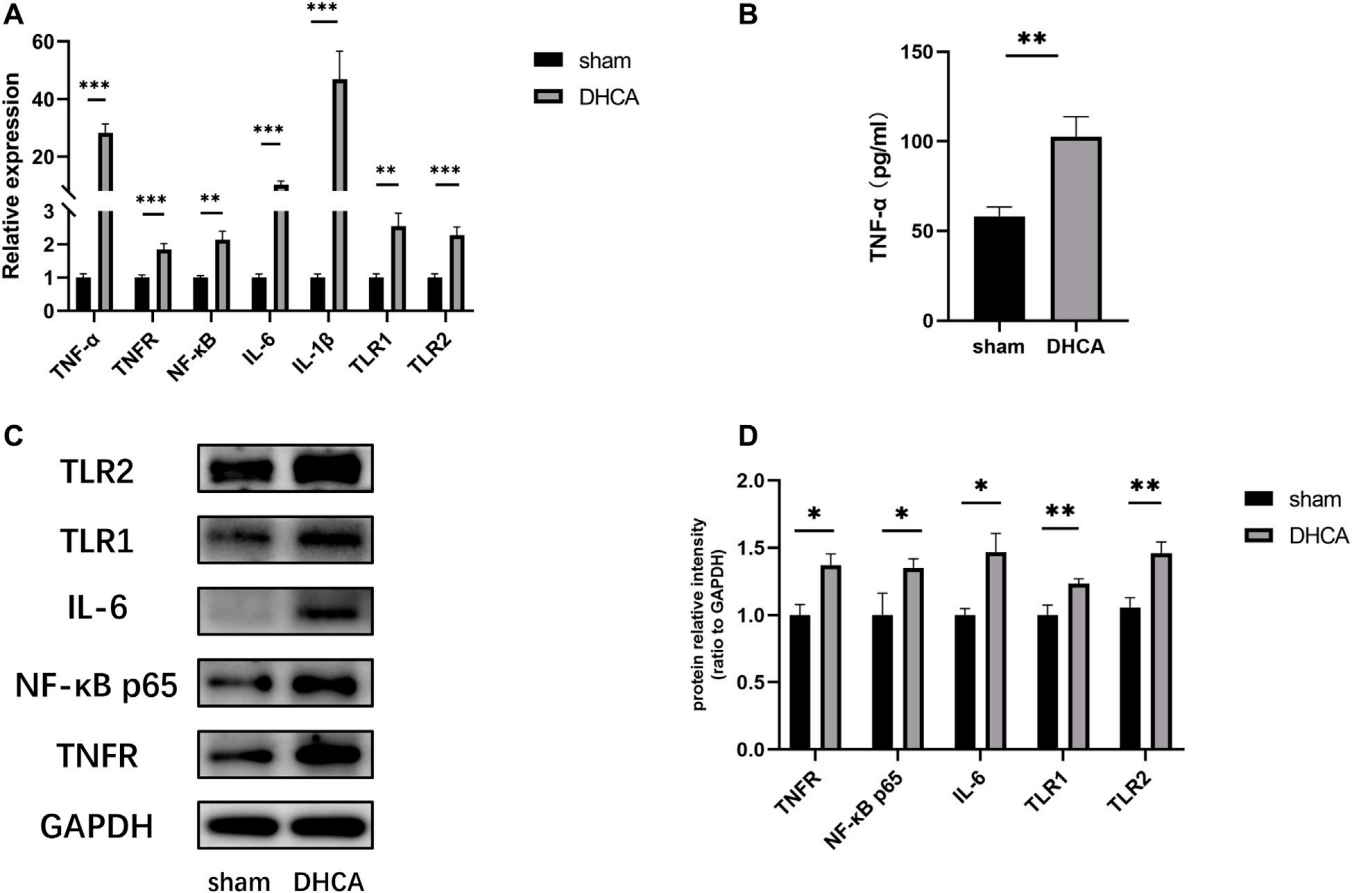
**(A)** The expressions of genes associated with the main pathways were validated by qRT-PCR. All reactions were repeated three times for each gene, GAPDH was used as an internal control, *n* = 5. **(B)** Compared with sham group, the TNF-α level in the cortex after DHCA was increased, *n* = 5. **(C,D)** The protein levels of genes associated with the main pathways after DHCA markedly increased compared with sham group, *n* = 5. **p < 0.01, ***p < 0.001, compared with sham group.

GO analyses were performed to investigate the functions of the DELs, including biological process (BP), cellular component (CC) and molecular function (MF), which may reveal the roles of the significantly differentially regulated genes. Furthermore, we found that some of the enriched functions and pathways were associated with DHCA. Figure 7A showed that the top 5 significant BP terms were extracellular stimulus, glucocorticoid stimulus, corticosteroid stimulus, cellular response to interleukin-6, and aging. The top 5 enriched CCs were implicated in extracellular space, hemoglobin complex, extracellular region part, extracellular region and C-fiber, while the significant MFs were oxygen transporter activity, oxygen binding, myosin tail binding, oxidoreduction-driven active transmembrane transporter activity and chemokine activity. Figure 7B showed the top 20 pathways revealed by the KEGG pathway analysis. The main significant pathways were related to tumor necrosis factor (TNF) signaling pathway, Malaria, NOD-like receptor signaling pathway, nuclear factor (NF) -kappa B signaling pathway and Toll-like receptor (TLR) signaling pathway. Simultaneously, the expressions of genes associated with these pathways, such as TNF-α, TNFR, NF-κ B, IL-6, IL-1β, TLR1, and TLR2, were increased after DHCA in cerebral cortex, as well as protein levels (Figure 8). Specific primers were designed as shown in Table 1.

### 3.5 Construction of the CeRNA Network

**FIGURE 9:**
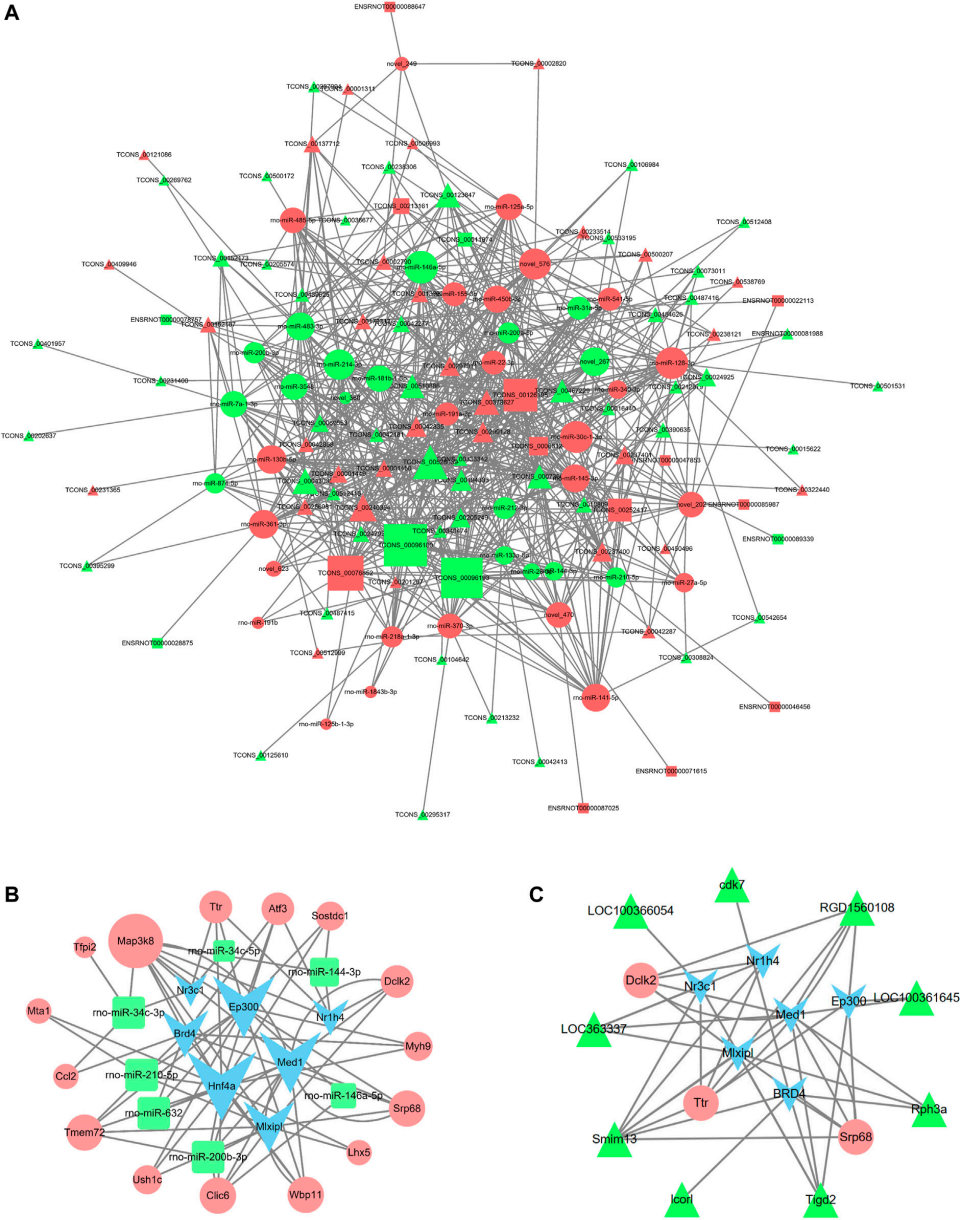
lncRNA-miRNA-mRNA coexpression network and TF-miRNA-mRNA network in DHCA. **(A)** The network displayed the correlations between the differentially expressed lncRNA-associate ceRNA profiles. The triangle nodes represent the lncRNAs, circle nodes represent the miRNAs, and rectangle nodes represent the mRNAs. The size of the node represents the degree centrality of the gene in the network. **(B,C)** The networks were built according to the TF-miRNA, TF-lncRNA, TF-mRNA, lncRNA-mRNA and miRNA-mRNA interactions. The blue nodes represent TFs, the green triangles represent lncRNAs, the green rectangles represent miRNAs and pink circles represent mRNAs. The size of the node represents the degree centrality of the gene in the network.

To further explore the potential lncRNA-miRNA-mRNA regulatory network, a coexpression analysis of differentially expressed lncRNA and mRNAs was conducted. We then identified lncRNA and miRNA interactions and miRNA and mRNA interactions. The last constructed lncRNA-miRNA-mRNA gene pairs included lncRNA as the decoy, miRNA as the core and mRNA as the target. A total of 223 lncRNA-mRNA pairs were obtained (PCC > 0.95 and *p* < 0.05), and 523 lncRNA-miRNA-mRNA targeted pairs were generated. The ceRNA network was shown in Figure 9A. For example, lncRNA Lcorl regulated Ttr by competing miRNA response elements of rno-miR-200a-3p.

### 3.6 Construction of the TF Regulatory Network

The regulatory networks of TF-miRNA-mRNA and TF-lncRNA-mRNA were established, as shown in Figures 9B,C, and it involved 7 TFs (Nr3c1, Ep300, Nr1h4, Med1, Mlxipl, Hnf4a, and BRD4), 9 lncRNAs (LOC363337, LOC100361645, LOC100366054, cdk7, RGD1560108, Rph3a, Tigd2, Lcorl, Smim13), 7 miRNAs (rno-miR-146a-5p, rno-miR-210-5p, rno-miR-200b-3p, rno-miR-34c-5p, rno-miR-34c-3p, rno-miR-632, and rno-miR-144-3p), and 15 mRNAs (Map3k8, Ttr, Atf3, Sostdc1, Dclk2, Myh9, Srp68, Lhx5, Wbp11, Clic6, Ush1c, Tmem72, Ccl2, Mta1, and Tfpi2). The regulatory network for rno-miR-200b-3p included 3 transcription factors: Hnf4a, Mlxipl, and Med1. Tmem72 was the common target gene that was coregulated by rno-miR-200b-3p and the four TFs Ep300, Hnf4a, BRD4, and Mlxipl. Here, rno-miR-210-5p and Hnf4a were found to coregulate the target gene Mta1.

## 4 Discussion

Although the mortality and morbidity after cardiovascular surgeries have been substantially improved, the utilization of DHCA can cause damage to multiple systems, including but not limited to the cardiovascular, kidney, and nervous systems (Cooper et al., 2000; Algra et al., 2014; Bartels et al., 2014; Fang et al., 2019). Stroke and temporary neurologic deficit were observed in 2 and 5.1% of patients undergoing aortic arch surgery using DHCA, respectively (Shi et al., 2020). The safe time range of DHCA is 20–30 min, and with the extension of DHCA time, the incidence of transient or persistent neurological dysfunction after DHCA surgery will significantly increase (Wei et al., 2015; Weber et al., 2019; Shi et al., 2020).

Many studies have suggested that the noncoding RNA family represented by lncRNAs and miRNAs plays a key regulatory role in the growth and differentiation of neurons, the development of the nervous system and the occurrence of neurological diseases (Liu et al., 2018; Mirzaei et al., 2018). Wei et al. found that miR-29 could reduce neuronal apoptosis and intracellular reactive oxygen species (ROS) by inhibiting the expression of the PUMA gene (Wei et al., 2018). miR-194-5p can directly inhibit the expression of the SUMO gene and promote the death of neurons (Datwyler et al., 2011). Zhang et al. reported that overexpression of the lncRNA Malat1 can reduce autophagy in vascular endothelial cells, thus reducing ischemic stroke injury (Zhang et al., 2017). However, studies on the regulatory role of ncRNAs in DHCA-induced organ injury have mainly focused on cardiovascular and renal aspects and less on the nervous system.

High-throughput sequencing is a second-generation sequencing method that can detect the expression of coding and noncoding RNAs in tissues (Chen et al., 2021). Its application is of great significance for understanding the regulation of basic biological systems and pathophysiological conditions and developing new treatments for many diseases (Khorkova et al., 2015). To date, few studies have focused on sequencing the expression profiles of the entire transcriptome and constructing a ceRNA regulator network to identify the core regulatory factors related to cerebral cortex injury following DHCA. Therefore, we performed RNA-seq for DHCA in a rat model to explore the key mechanisms underlying DHCA.

In this study, we screened the differential expression profiles between the DHCA and sham groups, and the results suggested 89 lncRNAs, 45 miRNAs and 59 mRNAs exhibit differential expressions. A total of 80 lncRNAs, 42 miRNAs and 18 mRNAs were found in the ceRNA networks. Through the GO enrichment analysis of DE profiles, the DELs were found to be mainly concentrated on responses to interleukin-6, regulation of nitric oxide biosynthetic processes and cellular responses to reactive oxygen species. These processes, which were reported in the previous literatures focused on cerebral ischemic insult, are mainly involved in cerebral inflammation and up-regulation of oxidative stress following ischemia-reperfusion injury (Wang et al., 2015; Wei et al., 2018). The current results of DELs were in line with our previous study, in which we found that Toll-like receptor 4 (TLR4), a key modulator of the pathway of TLR/NF-κB/MMP-9, could initialize the cascade of inflammatory molecules and cytokines, which results in degradation of tight junction proteins, therefore disrupts permeability of the BBB during DHCA (Tang et al., 2014). Furthermore, annotation of the DELs associated with the most significant KEGG pathways indicated the vital upstream pathways that regulate the inflammation and oxidative stress participate in the mechanism of the cerebral injury, such as the TNF signaling, Toll-like receptor signaling pathway and NF-κB signaling pathways. These involved pathways were partially verified by qRT-PCR results indicating the increased expression of related cytokines such as TNF-α, TNFR, NF-κB, IL-6 in the mouse DHCA model (Figure 8). There are growing evidences suggesting the noncoding RNAs are associated with molecular mechanisms of the inflammatory progress of the cerebral diseases (Jing et al., 2019), however, their roles in the inflammatory response during DHCA remains to be clarified (Yao et al., 2020).

Given the central positions the Inflammation related molecules occupy in the mechanism of the DHCA cerebral injury, we focused on screening candidate noncoding RNAs in the aggregate of current DELs. Of the up-regulating DELs, what interests us most is an inflammation related lncRNA Lcorl, which was reported to be linked to genes with known connections to AD pathophysiology and other neurodegenerative diseases (Scelsi et al., 2018), its attribution to inflammatory injury following DHCA is yet to be elucidated. Another previous study indicated that downregulated miR-200a could protect neural stem cells from cerebral infarction injury, possibly by regulating the STAT and MAPK/c-MYC signaling pathways (Ma et al., 2017). Additionally, miR-200a-3p and miR-200b-3p were implicated in the pathology of AD by suppressing the expression of β-amyloid protein precursor cleaving enzyme 1 and protein kinase A (Liu et al., 2015; Wang et al., 2019). In this study, T4 transporter transthyretin (Ttr) was predicted to be a common target of Lcorl and miR-200a-3p, was also reported to be strongly associated with amyloidosis and proteotoxicity(Tu et al., 2019). Azevedo et al. revealed that Ttr could activate microglia, leading to the secretion of TNF-α, IL-6 and nitric oxide, thus triggered neuronal damage (Azevedo et al., 2013). Therefore, we further selected the Lcorl-miR-200a-3p-Ttr pair of ceRNA for relative expression qRT-PCR verification. The result was consistent with the RNA-seq results, potentially indicated that Lcorl may regulate Ttr expression via sponging miR-200a-3p, in which further investigations are needed to elucidate the details of the regulatory networks.

Another noteworthy mRNA molecule which we found in the DEGs with highest fold change of expression is C-C motif chemokine ligand 2 (Ccl2). A previous study revealed that Ccl2 exhibited the highest levels of both mRNA and protein in an MCAO rat model and ameliorated ischemic lesions through binding the corresponding receptor CCR2 (Huang et al., 2018). Furthermore, Ccl2 was found to be associated with postischemic inflammatory responses, including transendothelial migration of monocytes and BBB disruption (Mahad et al., 2006; Roberts et al., 2012). However, the expression of Ccl2 and its mechanism in DHCA brain injury have not been reported. Among the TF-mRNA regulatory networks, bromodomain-containing protein 4 (BRD4), a member of the Bromo and Extra-Terminal (BET) family, was predicted to play a role in DHCA by binding to Ccl2. BRD4 expression was correlated with glial activation and cerebral I/R injury after MCAO in mice, and BRD4 suppression decreased the expression of pro-inflammatory factors by blocking NF-κB signaling, inflammasome activation and pyroptosis (Zhou et al., 2019). Therefore, our analysis further proved the upregulation of Ccl2 expression in the DHCA cerebral cortex, which is consistent with the study by Huang et al., and predicted that the pair of BRD4-Ccl2 may be a novel therapeutic target for DHCA brain injury. Based on the aforementioned analysis, the RNA-seq results of this study facilitated a comprehensive insight into these regulating networks in DHCA brain injury and provided evidence for potential molecular target of cerebral protection in DHCA.

Finally, we identified TF-regulatory networks composed of 7 TFs: BRD4, Ep300, Nr3c1, Mlxipl, Hnf4a, Nr1h4 and MED 1. As a transcription factor, MED1 is an important part of mega transcription factor-bound in trans enhancers, which can promote estrogen (E2)-mediated improvements in the transcriptional efficiency of downstream genes through ERα, thus promoting the growth of cancer cells (Liu et al., 2014). Estrogen can attenuate traumatic brain injury by inhibiting the activation of microglia and astrocyte-mediated inflammatory responses (Wang J. et al., 2020). In a previous study, Anet al. revealed that E-cadherin transcriptional activator (Ep300) could mediate brain-derived neurotrophic factor (BDNF) activation by targeting acetylated histones H3 and H4 on the BDNF promoter during the neuropathic pain associated with chronic constriction injury (Tan et al., 2020). According to the analysis results, we found that Ep300-miR-200b-3p-Tmem72 might play important role in DHCA cerebral injury, but the interactions among the aforementioned miRNAs, mRNAs and TFs warrants further exploration.

A large number of previous studies have found that there were differences in lncRNA expression in cerebrospinal fluid (CSF) and blood during the early phaseof diseases (Kraus et al., 2017; Taghizadeh et al., 2021). A study by Zhang et al. demonstrated that lncRNA RPL34-AS1 potentially serve as a valuable diagnostic and prognostic biomarker and therapeutic target in patients with brain glioma (Zhang et al., 2021). Measurement of lncRNAs levels in cerebrospinal fluid and leukocyte samples can be used as an ideal biomarker in different stages of Parkinson’s disease (PD) (Lv et al., 2020). Additionally, in a recent study Cai et al. showed downregulated lncRNA UCA1 ameliorates the damage of dopaminergic neurons in PD rats through the inhibition of the PI3K/Akt signaling pathway, which suggested that UCA1 might be a promising therapeutic target for PD (Cai et al., 2019). Based on the above findings, the clinical applications of lncRNA as novel tools for prediction and early diagnosis of cerebral diseases have gradually become a research spot lighting in recent years. Moreover, further investigation of molecular mechanism of lncRNAs is able to contribute to providing valuable clues of targets of treatment. Therefore, tracking lncRNAs in the central nervous system of patients with neurological dysfunction is of great value for prognosis, diagnosis and development of drugs.

Our study has some limitations. First, the transcriptome results in the current study reflect only the genetic changes in the acute phase of DHCA, and the transcriptome in a chronic model of DHCA may be quite different from the current results. Second, laboratory experiments to further verify these novel genes are lacking. Finally, using *p*-value instead of FDR inevitably cause slight loss of specificity and increase of false positive rate in the results of miRNAs.

In this study, the transcriptome related to cerebral injury in rats after DHCA was analyzed using high-throughput sequencing technology, and lncRNA-miRNA-mRNA and TF-miRNA-mRNA regulatory networks were constructed. Meanwhile we predicted that Lcorl-miR-200a-3p-Ttr, BRD4-Ccl2 and Ep300-miR-200b-3p-Tmem72 may participate in the pathogenesis of DHCA cerebral injury through multiple proinflammatory pathways. In summary, this study provides a direction for further investigation of changes in transcriptome of DHCA cerebral injury and clues of potential molecular targets for pharmaceutical research in the future.

## Data Availability

The datasets presented in this study can be found in online repositories. The names of the repository/repositories and accession number(s) can be found below: https://www.ncbi.nlm.nih.gov/genbank/, PRJNA739516.

## Supplementary Material

Supplementary Figure 1 The relative expression of differentially expressive RNAs. All reactions were repeated three times for each lncRNA **(A)**, miRNA **(B)** and mRNA **(C)**, GAPDH and U6 were used as an internal control, *n* = 5, * *p* < 0.05, ** *p* < 0.01, *** *p* < 0.001, compared with sham group.
